# Stochastic light concentration from 3D to 2D reveals ultraweak chemi- and bioluminescence

**DOI:** 10.1038/s41598-021-88091-0

**Published:** 2021-05-11

**Authors:** Ibtissame Khaoua, Guillaume Graciani, Andrey Kim, François Amblard

**Affiliations:** 1 Institute for Basic Science-Center for Soft and Living Matter, Ulsan, South Korea; 2 Department of Physics, University of Connecticut, Storrs, CT USA; 3 Department of Physics, Ulsan National Institute of Science and Technology, Ulsan, South Korea; 4 School of Life Sciences, Ulsan National Institute of Science and Technology, Ulsan, South Korea

**Keywords:** Applied optics, Imaging and sensing, Optical physics, Techniques and instrumentation

## Abstract

For countless applications in science and technology, light must be concentrated, and concentration is classically achieved with reflective and refractive elements. However, there is so far no efficient way, with a 2D detector, to detect photons produced inside an extended volume with a broad or isotropic angular distribution. Here, with theory and experiment, we propose to stochastically transform and concentrate a volume into a smaller surface, using a high-albedo Ulbricht cavity and a small exit orifice through cavity walls. A 3D gas of photons produced inside the cavity is transformed with a 50% number efficiency into a 2D Lambertian emitting orifice with maximal radiance and a much smaller size. With high-albedo quartz-powder cavity walls ($\rho =99.94\%$), the orifice area is $1/(1-\rho )\approx 1600$ times smaller than the walls’ area. When coupled to a detectivity-optimized photon-counter ($\mathcal{D}=0.015\,{\text{photon}}^{-1}\,{\text{s}}^{1/2}\text{ cm}$) the detection limit is $110\;{\text{photon}}\;{\text{s}}^{ - 1} \;{\text{L}}^{ - 1}$. Thanks to this unprecedented sensitivity, we could detect the luminescence produced by the non-catalytic disproportionation of hydrogen peroxide in pure water, which has not been observed so far. We could also detect the ultraweak bioluminescence produced by yeast cells at the onset of their growth. Our work opens new perspectives for studying ultraweak luminescence, and the concept of stochastic 3D/2D conjugation should help design novel light detection methods for large samples or diluted emitters.

## Introduction

How to best concentrate light from extended sources onto smaller surfaces is a long-standing challenge for a broad range of applications such as photo-voltaic converters, solar-powered furnaces, high-efficiency displays, or the concentration of light from a dim source onto a detector for imaging or non-imaging detection purposes. All passive solutions to these problems generally rely on the optimal combination of reflective and refractive elements that deterministically carry the light from the source to the target, with tentatively minimal losses caused by absorption, scattering, or aperture limitations. In this context, a fundamental law of geometrical optics, namely the conservation of the “optical étendue”, states that the optical radiance cannot increase between the source and the target. This law sets the maximal efficiency of optical concentration in reciprocal systems^1,2^. In the case of non-reciprocal or strongly scattering systems, very recent theoretical results suggest that the issue of maximal concentration efficiency could be addressed by the concept of “wave étendue”^3^. However, the very concept of étendue refers to a representation of the light as a 4D manifold in the phase space, that combines the 2D surface through which the light flows, with the 2D angular distribution of rays or waves over that surface. As a consequence and to the best of our knowledge, the light concentration problem has so far only been considered from the viewpoint of energy transfer between two surfaces, and it has no optimal solution when the light source extends in 3D.

**Figure 1:**
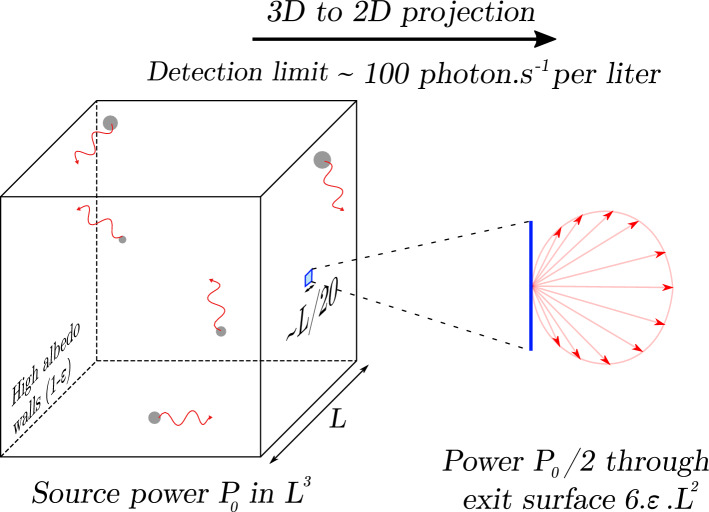
Optical projection and concentration from 3D to 2D. Stochastic conjugation of an extended 3D light source with a 2D emitter, using an Ulbricht (or integrating) cavity with diffuse reflective walls, and with a small exit orifice that act as a secondary Lambertian emitter. Optimal detection with a 2D detector coupled to the orifice is obtained when the ratio of the orifice area to the walls area equals the reflection loss coefficient $\epsilon = 1 - \rho$ , whereby 50% of the photons produced inside will exit the cavity. For a cubic emitter of with a volume $L^{3}$ and a square orifice $l^{2}$, the linear size of the 3D cavity is reduced to the linear size of the 2D orifice by the factor $l/L = \sqrt {6\epsilon }$, which can reach $1/20$.

The ultimate goal of the present work is the detection of ultraweak chemi- and bioluminescence emitted by extended 3D samples, when the density of photon emitters and their emission rate are extremely small. To improve the limit of detection in terms of emission rate density, we propose to concentrate this so-called “low-density” light by combining a 3D-to-2D dimensional projection with a reduction of the linear size. The 3D source is transformed into a 2D emitting surface by placing it inside an Ulbricht cavity^4^ with a small exit port and strongly scattering walls that provide a high Lambertian reflectivity. Due to multiple diffuse reflections on cavity walls, and under the provision that the source volume minimally absorbs the photons it produces, photons are stochastically and uniformly “focused” onto the exit port, which acts as a secondary 2D Lambertian emitter (Fig. 1). That secondary emitter can be then coupled to a sensitive detector. Ulbricht cavities, also called integrating spheres or cavities, have been used for the radiometry of non-homogeneous or non-isotropic light sources, for ring-down absorption spectroscopies, or for the purpose of generating spatially homogeneous sources, but never for the sake of optical concentration, to the best of our knowledge.

A statistical theory is presented here for high-albedo quartz-powder- or Teflon-based Ulbricht cavities, with wall reflectivity losses as low as $6 \times 10^{ - 4}$. In optimal conditions, the secondary Lambertian source typically emits 50% of the photons produced by the 3D sample, and its linear size can be as small as 18 times smaller than the linear size of the 3D source. This compression ratio of the linear size scales as the square root of the reflectivity loss coefficient of the walls. When coupled to a high-detectivity electron-multiplied charge-coupled device (EM-CCD, $\mathcal{D}=0.015\,{\text{photon}}^{-1}\,{\text{s}}^{1/2}\,\text{cm}$^5^), the limit of detection (LOD) for a signal-to-noise ratio (SNR) of 3, is a photon emission rate density in the order of ${\mathcal{J}}_{LOD}\approx 110\,\text{photon}\,{\text{s}}^{-1}\,{\text{L}}^{-1}$, i.e., $0.19\,\text{z}{\mathbb{E}}\,{\text{ L}}^{-1}\,{\text{ s}}^{-1}$ (units of zepto-Einstein, 1 $\text{z}{\mathbb{E}}={10}^{-21}\mathcal{N}$ photon).

As a proof of concept of our light concentration strategy, we report here on experiments designed to explore ultraweak chemiluminescence (CL) and bioluminescence (BL). The production of light by living organisms and by chemical reactions has been known for a very long time and used for a broad range of applications across various scientific and technological fields^6–14^, and CL and BL are generally thought to require catalytic or enzymatic activity respectively. However, numerous reports since the 1950s show that living cells and tissues in general spontaneously emit ultraweak light^15–23^, in the absence of any of the known light-producing enzymes, and probably as a consequence of the oxidative metabolic activity. It has also been known for decades that a large number of very simple oxidation and neutralization reactions in water do produce UV–visible photons with extremely small quantum efficiencies (${10}^{-12}$ to ${10}^{-15}$)^15,24,25^. This ultraweak CL is thought to be produced in the manner of a reverse photochemical reaction, by highly inefficient secondary reactions which branch on the main reaction, need oxygen, and produce free radicals that subsequently undergo radiative recombination^15,24–26^. However, ultraweak chemiluminescence largely remains a *terra incognita*, with little knowledge to connect the extremely inefficient radical-based radiative recombination processes to the core knowledge of photochemistry^27^, because of the daunting difficulty to detect ultraweak emission rate densities.

Experimental results are presented here for ultraweak CL and BL. First, we demonstrate that the disproportionation of hydrogen peroxide, which is one of the most important reactive oxygen species (ROS), does produce ultraweak light in pure water, i.e., in non-catalytic conditions. The estimated photon emission rate density in the sample is $72\,\text{z}{\mathbb{E}}\,{\text{L}}^{-1}\,{\text{s}}^{-1}$ for $[{\text{H}}_{2}{\text{O}}_{2}]=88\,{\text{mM}}$. Second, using the biological model of *S. cerevisiae* yeast cells in liquid culture, we find that cell growth is associated with an ultraweak luminescence peak that typically corresponds to $1.3\times 10^{-5}\,\text{photon}\,{\text{s}}^{-1}\text{ per cell}$. The novel light concentration scheme proposed here should be of general interested for a range of applications. Ulbricht cavities invented more than 100 years ago have been mainly used for radiometry application, but they could be very helpful to design novel light detection methods with unprecedented levels of sensitivity.

## Results

### Integrating sphere and optimal light collection

Since the pioneering work of Ulbricht more than 100 years ago^4^, we know the concept of Ulbricht cavity or sphere, also called integrating sphere, with inside walls covered with a strong Lambertian reflector. Any photon produced inside such a cavity is statistically reflected a very large number of times with a random direction at each reflection. This leads to a uniform irradiance on the cavity walls, be it a sphere or not. If a small exit hole is made through the walls, photons possibly find their way randomly through that exit. The exit probability relative to the probability of absorption by the walls is tuned by adjusting the exit surface area relative to the total area of the walls. In the following analysis, we consider a cavity with internal volume ${V}_{c}$ filled with a sample that uniformly produces photons with a rate density ${\mathcal{J}}_{s}$. Assuming that the cavity walls have a surface area ${\Sigma }_{c}$, with an exit port area ${\Sigma }_{h}=\beta {\Sigma }_{c}$, and a diffuse reflectance coefficient ${\rho }_{c}=1-{\epsilon }_{c}$ very close to unity, a simple probability argument gives the exit probability $f=\beta /({\epsilon }_{c}+{\rho }_{c}\beta )$. ${\rho }_{c}$ is called the albedo, with very small reflexion loss coefficient ${\epsilon }_{c}\ll 1$. The photon flux ${\phi }_{h}$ exiting the hole reads ${\phi }_{h}=f{\mathcal{J}}_{s}{V}_{c}/{\Sigma }_{h}$ and scales as $1/({\epsilon }_{c}+{\rho }_{c}\beta )$, leading to the following conundrum. While a smaller hole gives a larger flux that increases up to a finite limit proportional to $1/{\epsilon }_{c}$, it also leads to a vanishing exit probability, and no design optimization can be therefore made at that stage without an additional constraint. While the albedo ${\rho }_{c}$ is the most important optical property when the cavity is simply "filled" with vacuum, the picture is different when it contains a sample with an absorption length ${\mu }_{s}^{-1}$ and a volume ${V}_{s}={r}_{V}{V}_{c}$ enclosed in a surface area ${\Sigma }_{s}={r}_{\Sigma }{\Sigma }_{c}$, with $0<({r}_{V},{r}_{\Sigma })\le 1$ . The resulting transmission loss ${\epsilon }_{s}$ can easily be shown to amount ${\epsilon }_{s}={\mu }_{s}{L}_{c}[{r}_{V}/{r}_{\Sigma }]$, where ${L}_{c}=4{V}_{c}/{\Sigma }_{c}$ is the mean chord length of the cavity^28^. Since the additional losses caused by the sample simply add to the reflection losses, the effective albedo $\rho$ and the effective loss coefficient $\epsilon$ simply read: $\rho =1-\epsilon =1-[{\epsilon }_{c}+{\epsilon }_{s}]$. Moreover, ${\epsilon }_{c}$ depends on the wavelength, and ${\epsilon }_{s}$ on the absorption spectrum of the sample. However, although the following theory is based on considering $\epsilon (\lambda )\equiv \epsilon$ as a number and not a function, its results can be adapted for different wavelengths. Practically, only ${\epsilon }_{c}$ will be measured, and experiments will be interpreted assuming that $\epsilon \approx {\epsilon }_{c}$.

To maximize the detection of photons exiting the cavity, the exit port is conjugated with a region of the detector plane using a simple lens that maximizes the collection efficiency. The collection efficiency is given by the squared numerical aperture ${\text{NA}}^{2}$, while the magnification ratio $M$ determines the image area ${\Sigma }_{d}={\text{M}}^{2}{\Sigma }_{h}$ on the detector. The photon flux ${\phi }_{d}$ obtained on the detecting area then reads ${\phi }_{d}={\phi }_{h}({\text{NA}}/M{)}^{2}$. Under the assumption that the cavity is a cylinder with radius ${R}_{c}$ and height $2{R}_{c}$, we can compute the compression ratio between the cavity radius ${R}_{c}$ and the radius ${R}_{d}$ of the circular image on the detector, projected by the lens from the circular exit hole. The geometric compression ratio between these radii is given by:

1 $${R}_{d}={R}_{c}M\sqrt{6\epsilon /\rho }$$

### Noise model and detection limit

By definition of the detectivity $\mathcal{D}$ of the detector, the measurement of the flux ${\phi }_{d}$ using the area ${\Sigma }_{d}$ and exposure time $\tau$ comes with a signal-to-noise ratio given by:

2 $${\text{SNR}}=\mathcal{D}\sqrt{{\Sigma }_{d}}\sqrt{\tau }{\phi }_{d}$$

and we easily obtain:

3 $${\text{SNR}}=\mathcal{D}\sqrt{\tau }\left[\frac{\sqrt{\beta }}{\epsilon +\rho \beta }\right]\left[\frac{{\text{NA}}^{2}}{\text{M}}\right]\left[\frac{{V}_{c}}{{\sqrt{\Sigma }}_{c}}\right]{\mathcal{J}}_{s}$$

Each bracket in Eq. (3) essentially represents one of the three different contributions to the SNR and indicates how it can be separately optimized. The ratio $\left[\frac{\sqrt{\beta }}{\epsilon +\rho \beta }\right]$ reflects the trade-off for choosing the exit port area. It reaches a maximum for $\beta ={\Sigma }_{h}/{\Sigma }_{c}=\epsilon /\rho \approx \epsilon$, whereby $\left[\frac{\sqrt{\beta }}{\epsilon +\rho \beta }\right]=\frac{1}{2\sqrt{\epsilon \rho }}$. This optimum corresponds to the exit probability $f=1/2\rho \approx 1/2$. The ratio $\left[\frac{{\text{NA}}^{2}}{\text{M}}\right]$ will be optimized by choosing the best possible coupling lens. The last bracket is a squared length-scale, $\left[\frac{{V}_{c}}{{\sqrt{\Sigma }}_{c}}\right]={l}_{c}^{2}$ that characterizes the cavity geometry. For the cylindrical cavities used here, ${l}_{c}^{2}=\sqrt{2\pi /3}{R}_{c}^{2}\approx 1.4 {R}_{c}^{2}$. The quadratic term $\left[\frac{{V}_{c}}{{\sqrt{\Sigma }}_{c}}\right]={l}_{c}^{2}$ has the following meaning. If the linear size of all elements (the cavity, the lens and its focal length, and the detector) is increased by a given factor $\gamma$, the SNR then increases by a factor ${\gamma }^{2}$, provided the detector has a constant detectivity. This scaling is relevant to predict the expected performance with larger detectors or miniaturized set-ups. In optimal conditions, Eq. (3) can be simplified as:

4 $${\text{SNR}}={\mathcal{J}}_{s}\mathcal{D}\sqrt{\tau }\left[\frac{1}{2\sqrt{\rho \epsilon }}\right]\left[\frac{{\text{NA}}^{2}}{\text{M}}\right]{l}_{c}^{2}$$

To evaluate how much the SNR benefits from the integrating cavity strategy, a simple comparison can be made using the following assumptions: the sample is a sphere of radius ${R}_{c}$, and all the photons produced inside it are considered to originate from an equatorial disk conjugated to the detector with the same numerical aperture and magnification. If we roughly overestimate that the detector receives a proportion ${\text{NA}}^{2}/2$ of the photons produced by the sphere, the SNR can be overestimated ${\text{SNR}}={\mathcal{J}}_{s}\mathcal{D}{l}_{c}^{2}\sqrt{\tau }\left[\frac{{\text{NA}}^{2}}{\text{M}}\right]$. The comparison with Eq. (4) shows that the integrating cavity enhances the SNR at least by a factor $1/2\sqrt{\epsilon }$.

Practically however, the choice of a high detectivity detector is much more constrained than the choice of cavities that can be easily customized. Therefore, the scale ${l}_{c}$ defined by the geometry of the cavity is not what should be primarily decided when designing the experiment, and the scale given by the detection area ${\Sigma }_{d}$ is much more relevant. Using ${\Sigma }_{d}$ instead as the master scale, the optimal cavity volume is

5 $${V}_{c}={\left[\frac{{\Sigma }_{d}}{\epsilon }\right]}^{3/2}\frac{{\rho }^{3/2}}{3\sqrt{6\pi }{\text{M}}^{3}}$$

and the SNR then reads

6 $${\text{SNR}}={\mathcal{J}}_{s}\mathcal{D}\sqrt{\tau }{\Sigma }_{d}\frac{1}{3\sqrt{6\pi }}\left[\frac{{\text{NA}}^{2}}{2}\right]\left[\frac{{\rho }^{1/2}}{{\epsilon }^{3/2}{\text{M}}^{3}}\right]$$

or

7 $$\text{SNR}={\mathcal{I}}_{s}\mathcal{D}\sqrt{\tau /{\Sigma }_{d}}\left[\frac{{\text{NA}}^{2}}{2\rho }\right]$$

where ${\mathcal{I}}_{s}$ is the emission rate ${\mathcal{I}}_{s}={\mathcal{J}}_{s}{V}_{c}$ integrated over the cavity volume in units of $\text{photon}.{\text{s}}^{-1}$.

Altogether the above analysis can be summarized as follows. If a detector with a detectivity $\mathcal{D}$ and a detection area ${\Sigma }_{d}$, is coupled to the exit port of an integrating cavity through a lens with numerical aperture ${\text{NA}}$ and a magnification ${\text{M}}$, the SNR produced by a photon emission rate $\mathcal{I}$ or rate density $\mathcal{J}$ inside the cavity is given by Eqs. (7) and (6), provided the cavity has an exit port area set as ${\Sigma }_{h}={\Sigma }_{d}/{\text{M}}^{2}$ and a volume obeying Eq. (5). In these conditions, the limit of detection defined by $\text{SNR}=3$ can be expressed in term of the limit photon emission rate ${\mathcal{I}}_{\text{LOD}}$ or rate density ${\mathcal{J}}_{\text{LOD}}$ by:

8 $${\mathcal{I}}_{\text{LOD} }=3{\mathcal{D}}^{-1}\sqrt{{\Sigma }_{d}/}\tau \left[\frac{2\rho }{{\text{NA}}^{2}}\right]\,\text{and}\,{\mathcal{J}}_{\text{LOD}}={\mathcal{I}}_{\text{LOD} }/{V}_{c}$$

### Practical design for optimal volumetric photon detection

**Figure 2:**
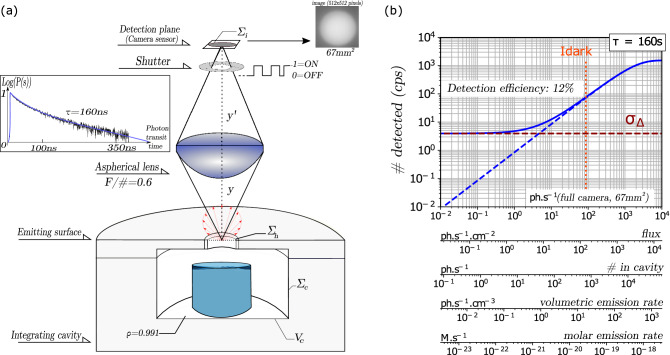
Optical set-up and expected performances. Integrating cavity and optical detection set-up. The set-up (**a**) is made of a cylindrical integrating cavity, a high numerical aperture lens (${\text{NA}}=0.29$), a cooled EM-CCD^5^, and the whole set-up is assembled in a light-proof enclosure (see “Methods”). Cavities made of Teflon or compressed quartz powder had the same 3.8 cm diameter and height, and ${V}_{C}=43\,{\text{cm}}^{3}$ volume. The intracavity albedo $\rho$ is assessed from the exponential time-decay P(s) of a short light pulse inside the cavity (insert to (**a**)). $\rho =1-\epsilon =0.991$ for Teflon and $0.9994$ for quartz. The circular exit port area ${\Sigma }_{h}=0.6\,{\text{cm}}^{2}$ matches the fraction $\epsilon /\rho$ of the internal area ${\Sigma }_{c}$, i.e., $0.9\%$ for Teflon and $0.06\%$ for quartz. The exit port is imaged on the detector with a magnification $M=0.94$ using a large lens (${\text{NA}}=0.29$, $\upphi =40\text{ mm}$). With the optimal port area, the photon exit probability is $1/2\rho \approx 50\%$ for any albedo. The probability to reach the detector from within the cavity is ${\text{NA}}^{2}/2\rho \approx 4.2\%$ whatever the albedo. Under optimal conditions with a fixed hole area, a larger reflectivity corresponds to a larger cavity area and volume. The EM-CCD detector receives the signal on a ${\Sigma }_{d}=0.46\,{\text{cm}}^{2}$ circular image detection area. Background subtraction between successive frames is done with a shutter. Assuming maximal detection quantum efficiency ($0.9$), (**b**) shows the counts per second (cps) on the detector area ${\Sigma }_{d}$ as a function of the incident photon rate. The overall detection probability is $4.2\%$ QE ≈ $3.8\%$. Under optimal exposure *τ*_*opt*_ = 160 s^5^, the noise level ${\sigma }_{\Delta }$ corresponds to $3.25\,{\text{counts}} \, {\text{s}}^{-1}$ (red dotted line). The level ($3{\sigma }_{\Delta }$) is defined as the limit of detection (LOD) defined by SNR = 3. Additional axes show how the incident photon rate on the detection area translates, into (from top to bottom): the incident flux on the detector, the photon production rate inside the Teflon cavity, the corresponding rate density (volumetric emission rate) in ${\text{photon}}\,{\text{s}}^{-1}\,{\text{cm}}^{-3}$, and rate density expressed in Molar per second, i.e., moles of photons (Einstein) per liter per second or (${\mathbb{E}}\,{\text{L}}^{-1}\,{\text{s}}^{-1}$). Since the ${\text{LOD}}$ is enhanced by a factor $[{\epsilon }_{quartz}/{\epsilon }_{Teflon}{]}^{-3/2}={15}^{3/2}\approx 60$ for quartz compared to Teflon cavities, the last 3 scales should be shifted accordingly for quartz, with a detection limit of $0.19\,\text{z}{\mathbb{E}}\,{\text{L}}^{-1}\,{\text{s}}^{-1}$ for quartz instead of $11\,\text{z}{\mathbb{E}}\,{\text{L}}^{-1}\,{\text{s}}^{-1}$ for Teflon.

Following the prescriptions of the above theoretical analysis, an integrating cavity was designed as a cylinder, with inside diameter $2{R}_{C}$ and height $h$ both equal to $3.8\,\text{cm}$, and with 2.5 cm thick Teflon walls (Fig. 2a). We also built cavities with walls made of compressed quartz powder^29^. Using the pulse stretching method^30^ the albedo $\rho$ was measured to be ${\rho }_{\text{Teflon}}=0.991$ and ${\rho }_{\text{quartz}}=0.9994$, which corresponds to loss coefficients $\epsilon_{{{\text{Teflon}}}} = 9 \times 10^{ - 3}$ and $\epsilon_{{{\text{quartz}}}} = 6 \times 10^{ - 4}$. For Teflon cavities, the exit port area ${\Sigma }_{h}$ was set as the fraction $\epsilon /\rho \approx 0.9\%$ of the inside cavity surface area. The coupling lens, ${\text{NA}}=0.29$, used with no magnification (${\text{M}}=0.94$), produced a $0.46\,{\text{cm}}^{2}$ disk-shape image on the detector plane (Fig. 2a). In this configuration, the flux on the detector typically represents $({\text{NA}}/{\text{M}}{)}^{2}\approx 9.5\%$ of the flux at the exit port. The overall photon collection efficiency given by the product of the cavity exit probability $f=1/2\rho$ by the lens collection efficiency ${\text{NA}}^{2}$ amounts $4.2\%$.

Finally, the detector is a cooled EMCCD with a $0.67\,{\text{cm}}^{2}$ area and ${512}^{2}$ pixels operated in the binary photon counting mode. Pixels delivered a single-bit count, 0 or 1, with a quantum efficiency close to unity ($QE\approx 0.9$)^5^. As described by Khaoua et al.^5^, the optimal operation of this camera is based on measuring the signal as a difference within pairs of successive acquisitions made by opening/closing the camera shutter, and with an optimal exposure time ${\tau }_{opt}=160\text{ s}$. In such conditions, the noise level is the standard deviation ${\sigma }_{\Delta }$ measured in the absence of signal, and the detectivity was found to be $\mathcal{D}=0.015\,{\text{photon}}^{-1}\,{\text{s}}^{1/2}\,{\text{cm}}^{1}$^5^. The noise ${\sigma }_{\Delta }$ scales as the square root of the detection area, and it reaches ${\sigma }_{\Delta }=520\,{\text{counts}}$ for a surface area ${\Sigma }_{d}=0.46\,{\text{cm}}^{2}$. Altogether, for Teflon cavities, Eq. (4) numerically writes

9 $${\text{SNR}}=3.6{10}^{-2} {\stackrel{\sim }{\mathcal{J}}}_{s}\sqrt{\stackrel{\sim }{\tau }}$$

where ${\stackrel{\sim }{\mathcal{J}}}_{s}$ and $\stackrel{\sim }{\tau }$ are the dimensionless values of ${\mathcal{J}}_{s}$ and $\tau$ obtained considering basic units of second and centimeter. From Eq. (9) and for $\stackrel{\sim }{\tau }=160$, we find that ${\text{SNR}}=1$ is obtained for a critical photon emission rate density ${\mathcal{J}}_{s}=2.2 \,\text{photon}\,{\text{s}}^{-1}\,{\text{cm}}^{-3}$, which corresponds to $3.6 \times 10^{ - 21}$ moles or $3.6$ zepto-moles of photons (z ${\mathbb{E}}$) per liter per second: ${\mathcal{J}}_{s}=3.6\,\text{z}{\mathbb{E}}\,{\text{L}}^{-1}\,{\text{s}}^{-1}$. On the detector, this corresponds to $4.0\,\text{photon}\,{\text{s}}^{-1}$ and ${\phi }_{d}=8.6\,\text{photon}\,{\text{s}}^{-1}\,{\text{cm}}^{-2}$. The limit of detection inside the cavity is given by the emission rate ${\mathcal{I}}_{\text{LOD-Teflon}}\approx 280\,\text{photon}\,{\text{s}}^{-1}$ or the rate density ${\mathcal{J}}_{\text{LOD-Teflon}}\approx 6.6\,\text{photon}\,{\text{s}}^{-1}\,{\text{cm}}^{-3}$, which is equivalent to $11\,\text{z}{\mathbb{E}}\,{\text{L}}^{-1}\,{\text{s}}^{-1}$. Those results are illustrated by Fig. 2b, showing the count rate on the detector as a function of rate of photons arriving on the detection area for the maximal quantum efficiency $\text{QE}\approx 0.9$. We also illustrate the relation between the various scales of interest, from the photon rate on the detector to the molar emission rate in the cavity expressed in ${\mathbb{E}}\,{\text{L}}^{-1}\,{\text{s}}^{-1}$. For quartz-powder cavities, Eq. (5) indicates that the optimal volume is $[{\epsilon }_{\text{quartz}}/{\epsilon }_{\text{quartz}}{]}^{-3/2}={15}^{3/2}=58$ times larger than for Teflon. As a consequence, we expect a $58$ times smaller limit of detection for quartz, with ${\mathcal{J}}_{\text{LOD-quartz}}\approx 0.11\,\text{photon}\,{\text{s}}^{-1}\,{\text{cm}}^{-3}$ or $0.19\,\text{z}{\mathbb{E}}\,{\text{L}}^{-1}\,{\text{s}}^{-1}$ (Fig. 2b).

### Chemiluminescence of the catalyzed disproportionation of H_2_O_2_

Ultraweak chemiluminescence (CL) and bioluminescence (BL) are fundamentally related, because some of the free radicals involved in CL^15,26,31–33^ are also essential for biological processes such as single-electron transfer, oxidative phosphorylation, redox homeostasis, oxidative stress, or cell signaling^34^. Among them, hydrogen peroxide (${\text{H}}_{2}{\text{O}}_{2}$) is a radical-based reactive oxygen species (ROS) that plays a key role in both chemistry and biology, most likely because it is much more stable compared to other ROS. In particular, while ${\text{H}}_{2}{\text{O}}_{2}$ acts as biological messenger for many cellular responses, it is also involved in the oxidative stress response which comes with toxic effects, and it is enzymatically degraded by catalase or myeloperoxidase during detoxification processes^34,35^. While non-enzymatic degradation also occurs in aqueous solution by the luminescent metal-catalyzed Fenton reaction, or through the radical-mediated oxidation of various anions or proteins^15,26,32,34^, one single report shows evidence that the non-catalytic disproportionation of ${\text{H}}_{2}{\text{O}}_{2}$ in salt solutions produces a very weak chemiluminescence^36^. This luminescence is most likely due to electronic transitions of singlet oxygen ${}^{1}{\text{O}}_{2}$^26,36,37^. The fundamental question therefore stands out, to know if ${\text{H}}_{2}{\text{O}}_{2}$ disproportionation is luminescent in pure water?

**Figure 3:**
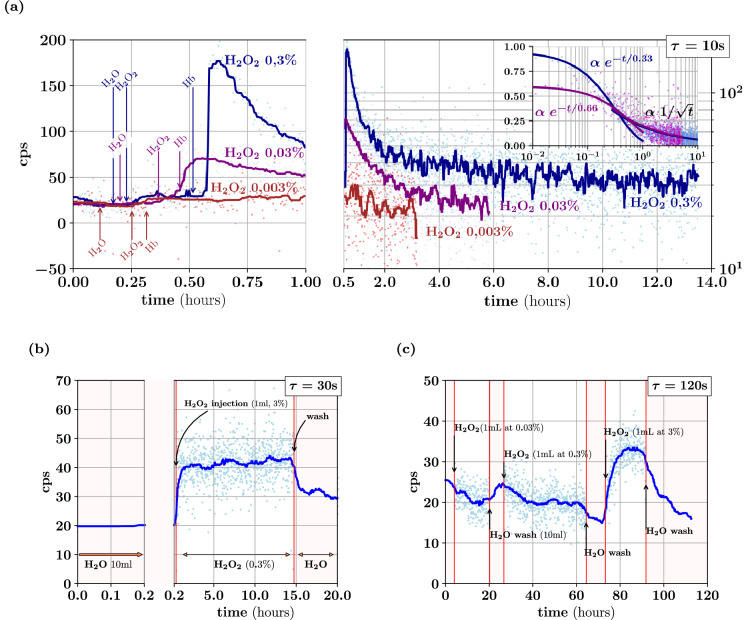
Luminescence from non-catalytic disproportionation of H_2_O_2_. Luminescence of ${\text{H}}_{2}{\text{O}}_{2}$ in water, with or without catalyst. (**a**) Shows the count rate (counts per second, cps) measured on the detector for a *τ* = 10 s exposure time along 3 different procedures (bleu, cyan, and red) in which 1 mL of ${\text{H}}_{2}{\text{O}}_{2}$ is injected in 10 mL of water, followed by 1 mL of hemoglobin (0.5 mg mL^−1^), to reach a final concentration of $0.3\%$, $0.03\%$, or $0.003\,{\text{wt}}\%$ respectively. Two subplots of the same data are presented to separately show the initial and the long time-scale behavior. Dots represent the value measured for each frame, while lines indicate the moving median value over 7 frames. The insert shows how the data could be fit, initially with an exponential, and with a power law at later times. (**b**) Count rate observed over *τ* = 30 s in the absence of hemoglobin in 10 mL of pure water upon the injection 1 mL of ${\text{H}}_{2}{\text{O}}_{2}$ to reach a final $0.03\%$ concentration. After 15 h, the medium is washed away and replaced with the same volume of water. Two adjacents subplots of the same data illustrate the initial and the long time-scale behavior. (**c**) Count rates observed for a *τ*_*opt*_ = 120 s exposure time in the absence of hemoglobin in 10 mL of pure water upon the successive injection 1 mL of ${\text{H}}_{2}{\text{O}}_{2}$ at increasing concentrations, to reach $0.003\%$, $0.03\%$ and finally $0.3\%$. The medium is washed away and replaced with the same volume of water between the injections. All experiments were repeated at least twice with each detector and integrating cavities. Typical time traces are shown.

In a first series of experiments, we measured the luminescence produced by ${\text{H}}_{2}{\text{O}}_{2}$ in the presence of 0.5 mg mL^−1^ hemoglobin. This observation is congruent with previous reports^8,38,39^. When $[{\text{H}}_{2}{\text{O}}_{2}]=88\,{\text{mM}}$ ($0.3\,{\text{wt}}\%$), the luminescence typically decays exponentially over one hour (Fig. 3a). The wavelength of this luminescence is not measured here. However, to estimate the photon emission rate density in the solution, we assumed that the wavelength of emitted photons corresponds to a $0.9$ quantum efficiency (${\text{QE}}$). In fact, $0.8\le {\text{QE}}(\lambda )\le 0.9$ for $400\,{\text{nm}}\le \lambda \le 750\,\text{nm}$^5^. The luminescence peak corresponds to a typical photon emission rate of $4 \times 10^{3} \;{\text{photon}}\;{\text{s}}^{ - 1}$ in the cavity. Considering the cavity volume, this peak approximately corresponds to $93\,\text{photon}\,{\text{cm}}^{-3}\,{\text{s}}^{-1}$ or $160\,\text{z}{\mathbb{E}}\,{\text{L}}^{-1}\,{\text{s}}^{-1}$ with ${\text{SNR}}\approx 11$. Considering the 11 mL sample volume instead, the actual sample emission rate density typically amounts to $620\,\text{z}{\mathbb{E}}\,{\text{L}}^{-1}\,{\text{s}}^{-1}$. From the estimated total number of photons produced by the reaction (5–10 × 10^6^ photons) and the initial number of ${\text{H}}_{2} {\text{O}}_{2}$ molecules (5.3 × 10^20^), the ratio gives an estimated luminescence quantum yield $Y \approx$ to 2 × 10^−14^.

### Non-catalytic luminescence from H_2_O_2_ disproportionation

In a second series of experiments, we assessed the residual luminescence produced by $[{\text{H}}_{2}{\text{O}}_{2}]=88\,{\text{mM}}$ in pure water in the absence of hemoglobin. Figure 3b clearly shows a steady-state production of light detected with a slightly larger exposure time *τ* = 30 s. It is important to note that no luminescence is detected when the cavity is washed, and the sample is replaced by pure water. The steady-state count rate on the detector typically exceeds the background by 20 counts per second and corresponds to $480\,{\text{photon}}\,{\text{s}}^{-1}$ being produced by the sample. This corresponds to an effective rate density of approximately $19\,\text{z}{\mathbb{E}}\,{\text{L}}^{-1}\,{\text{s}}^{-1}$ in the cavity. This rate is twice the LOD in Teflon cavities, and it would be $100$ times the LOD in quartz cavities. Since the sample volume is smaller that the cavity, the actual rate density in the sample is ${\mathcal{J}}_{{\text{H}}_{2}{\text{O}}_{2}}\approx 73\,\text{z}{\mathbb{E}}\,{\text{L}}^{-1}\,{\text{s}}^{-1}$. These estimates are again based on considering an emission spectrum such that ${\text{QE}}\approx0.9$. Assuming a first-order luminescence scheme ${\mathcal{J}}_{{\text{H}}_{2}{\text{O}}_{2}}={k}^{+} Y [{\text{H}}_{2}{\text{O}}_{2}]$, the emission rate constant, i.e., the product of the reaction rate ${k}^{+}$ and emission quantum yield $Y$, can be estimated as $k^{ + } Y \approx 8 \times 10^{ - 19} \;{\text{s}}^{ - 1}$. When the concentration of hydrogen peroxide is increased stepwise, with $[{\text{H}}_{2}{\text{O}}_{2}]=0.88$, $8.8$, and $88\,{\text{mM}}$, the luminescence signal exceeds the detection limit when reaching the highest concentration (Fig. 3c).

### Ultraweak bioluminescence of yeast liquid cultures

**Figure 4:**
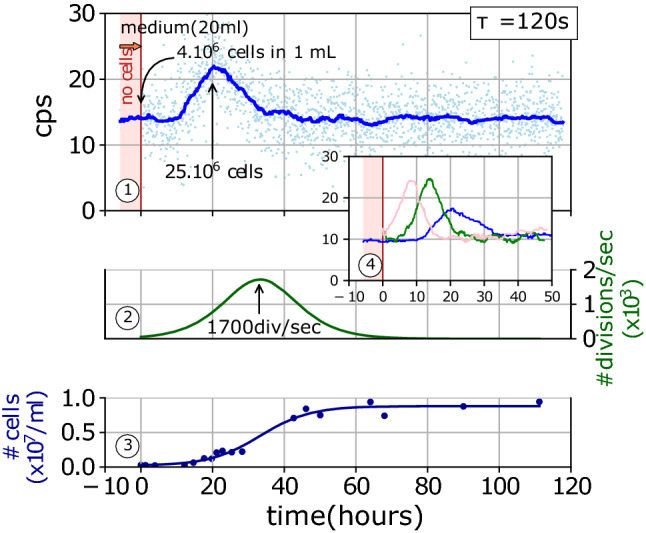
Spontaneous bioluminescence of yeast. Spontaneous luminescence of yeasts growing in liquid culture. Graph no. 1 shows the spontaneous luminescence of yeasts growing in a glucose medium (20 mL). Dots represent the measurement from individual frames and the solid line shows the moving median value over 15 frames. The signal obtained prior to cell seeding indicates the background level. The growth is monitored by counting cells manually (Graph no. 3, blue dots and blue line fit), and the division rate (Graph no. 2) is computed as the time-derivative of the fit of Graph no. 3. Graph no. 4 shows the extent of reproducibility of the experiment with the same BY4742 strain growing in similar conditions, with two similar cameras and a similar integrating cavity.

In this last section, we used our detection method to examine the ultraweak luminescence spontaneously produced by yeast cells. Because yeasts belong to lower eukaryotes, it represents a popular model to investigate a number of phenomena of eukaryotic cell biology, including energy metabolism, oxidative respiration, or the response to oxidative stress. In that respect, it is not surprising that a few yeast strains have been already investigated and shown to spontaneously produce ultraweak luminescence detected in the 200–650 nm wavelength region, possibly with an emission peak at 540 nm potentially related to lipid peroxidation^15,17,18^. Using the BY4742 strain of *S. cerevisiae* in aerobic conditions with defined medium containing glucose, we grew asynchronous cultures from stationary phase inocula, by seeding $$4 \times 10^{6}$$ cells in 1 mL into a 20 mL sample. $$10$$ h after seeding, luminescence was detected, which peaked $$10$$ h later and decreased almost symmetrically (Fig. 4). The peak emission approximately corresponds to $$8$$ counts per s. Assuming again that $${\text{QE}}\approx 0.9$$, which is a reasonable assumption given the 540 nm peak mentioned above, the luminescence peak corresponds approximately to a $$210\,\text{photon}\,{\text{s}}^{-1}$$ output in the cell culture, while the integrated luminescence peak integrated over time can be estimated to carry $$9 \times 10^{6}$$$$\text{photons}$$. From fitting the growth curve (Fig. 4), the overall growth rate was seen to peak $$33$$ h after seeding (Fig. 4), and we found an estimated division time of $$6 {\text{h}}$$. Obviously, the luminescence maximum was not associated with the peak of the division activity which came $$13$$ h later, but rather with the onset of the growth phase. More precisely, at the luminescence peak, the cell density was approximately $$10\%$$ of the stationary density, with 2 × 10^7^ cells in the sample. It was beyond the scope of the present work to further analyze how the dynamics of light emission is related to the dynamics of yeast, but a pattern of ultraweak luminescence was reproducibly observed to be associated with the initiation of the growth process (Fig. 4), with a peak emission that corresponds to $$\approx 1.3\times10^{ - 5} \;{\text{photon}}\;{\text{s}}^{ - 1} \;{\text{cell}}^{ - 1}$$.

## Discussion

### Detection of ultraweak light from “dilute” photon sources

Since their invention by Ulbricht^4^, diffuse reflectance integrating cavities have mostly been used in radiometry to quantify the total radiant flux from extended or non-collimated sources. Because of the ring-down effect due to multiple Lambertian reflections, these cavities increase the path length of light and uniquely enable very sensitive and absolute measurements of spectroscopic properties such as luminescence quantum yield, or absorption or scattering cross-sections^30,40–43^. More recently, we used a quartz powder cavity as a random amplifier with a highly coherent light source to build a very sensitive dynamic interferometer^44^. However, to the best of our knowledge, while diffuse cavities have been used only once for a fluorescence assay with a sensitivity improvement down to the $500\,\text{fM}$ level using the quartz powder material used here^45^, they have are not been used to improve the detection limit of bio- or chemiluminescence instruments.

The motivation of the present work was to explore, with absolute measurements and the best possible sensitivity, the ultraweak bioluminescence spontaneously produced by living cells, and the chemiluminescence of the non-catalytic disproportionation of ${\text{H}}_{2}{\text{O}}_{2}$. On this path, we addressed the relatively uncharted challenge to quantify extremely small photon emission rate densities produced by extended 3D samples, or highly “dilute” photon sources. In such circumstances, the lack of information about the position of emitters makes it impossible to efficiently bring photons from a large 3D volume onto a relatively smaller 2D detector, and the reason is very simple. Indeed, using classical refractive or reflective optical elements like lenses or mirrors, it is impossible to optically conjugate a volume with a surface. To reduce the considerable losses incurred by the necessarily poor “impossible conjugation” between a volume and a surface, lensless devices have been proposed, in which the sample is practically sitting on the detector window^7,11^. Various mirror-based configurations have also been used^9^. These strategies do improve light collection, but the 3D/2D issue with conjugation is not addressed. Meanwhile, they raise a serious issue with the SNR, because a reasonable aperture is only obtained if the detector and the sample practically have the same linear size, leading to the choice between a large detector noise or a small signal. This conundrum has no solution but one: light emission must be both dimensionally reduced from 3 to 2D, and concentrated in linear size with minimal losses (Fig. 1 and Supplementary Fig. 1).

### Light compression from 3 to 2D

Our main contribution here, with a simple theoretical model and experiments, is to enhance the detection sensitivity by compressing the linear size and reducing the dimension of the sample light source from a large 3D volume to a much smaller 2D surface. Quantitatively, using square-cylindrical cavities ($h=2{R}_{c}$) with a circular detection zone with radius ${R}_{d}$ and the uniquely small reflection losses (${\epsilon }_{quartz}=6$ × 10^−4^) of compressed quartz powder walls invented recently^29^, the optimal linear size-compression ratio ${R}_{c}/{R}_{d}$ given by Eq. (1) is a factor $\approx 18$. The theory shows that this ratio scales as ${\epsilon }^{-1/2}$ and is equal to $4.5$ for Teflon (${\epsilon }_{Teflon}=9$ × 10^−3^). As a result, the large 3D actual sample is virtually replaced by an effective 2D source that sits on the surface of the cavity exit port and behaves as a flat Lambertian emitter. In optimal conditions, this effective source emits the fraction $1/2\rho \approx 50\%$ of the photons produced inside the cavity. Interestingly, because the randomness of reflections inside the cavity, the effective 2D sample emits the same Lambertian flux regardless of its shape, and it could be shaped for instance as a narrow slit for the sake of ultra-sensitive spectroscopy.

We combined this principle of size compression and dimensional reduction of light, with a detectivity-optimized EM-CCD recently described in a companion paper^5^. What matters most here is not the noise level per se, but how it scales with the exposure time and the detector surface, and how to maximize the time-density of signal information. We show that our EM-CCD operated in the photon counting mode compares quite favorably with the detectivity of the best cooled PMT^5^. In addition, the “corners” of the image (Fig. 2a) could be used as an internal reference to monitor the stray light and estimate its fluctuations on the flight. Using this combination we could detect extremely small photon emission rate densities, with a detection limit of ${\mathcal{J}}_{\text{LOD-quartz}}\approx 0.11\,\text{photon}\,{\text{s}}^{-1}\,{\text{cm}}^{-3}$ and ${\mathcal{J}}_{\text{LOD-Teflon}}\approx 6.6\,\text{photon}\,{\text{s}}^{-1}\,{\text{cm}}^{-3}$ for ${\text{SNR}}=3$. For red photons ($\lambda =600\text{ nm}$) in quartz cavities, the limit corresponds to ${\mathcal{J}}_{\text{LOD-quartz}}\approx 40\,\text{zeptoW}\,{\text{cm}}^{-3}$.

### Limits of the enzymatic of ATP and bacteria

At this point, we were frustrated to realize that the papers on luminescence-based detection methods rarely report on light signals in absolute numbers of photons, and never report on detection limits (LOD) in units of the density of photon emission rate^7,14,46^, thus precluding any direct sensitivity comparison with the present work. However, knowing that the luciferase-based luminescence detection of ATP has been optimized up to the detection of one single bacterium typically in 0.1 cm^3^ luminometer samples^13^, this current detection limit can be translated into an order of magnitude photon emission rate density as follows. Given that (a) one bacterium typically contains ${10}^{-18}\mathcal{N}\approx 6$ × 10^5^ ATP molecules which corresponds to $[\text{ATP}]\approx 10\,\text{fM}$ in the reaction medium, that (b) luciferase typically produces $1$ photon for $2$ ATP molecules^47^, and assuming that (c) detection can be achieved in $1$ minute, we infer that the order of magnitude of the emission rate from the ATP molecules of one bacterium in a 0.1 cm^3^ sample is $\mathcal{I}\approx 5$ × 10^3^ photon s^−1^. A similar order of magnitude can be inferred from the known detection limit (${10}^{-20}$ moles in 0.1 cm^3^) of luciferase molecules (Luciferase assay system, Promega) or horseradish peroxidase^20^. By comparison with our limit of detection of ${\mathcal{I}}_{\text{LOD}}\approx 280\,\text{photon}\,{\text{s}}^{-1}$ inside the cavity, the emission rate estimated above is $17$ larger and it should be easily detected with $\text{SNR}\approx 50$ using our method.

But the most important point here is that this larger SNR value does not require to concentrate the photon emitters inside the small 0.1 cm^3^ volume of classical luminometer samples. Instead, the very small limit of detection is obtained from a $430$ times larger sample with 43 cm^3^ Teflon cavities, and with an additional factor $[{\epsilon }_{quartz}/{\epsilon }_{Teflon}{]}^{-3/2}=58$ for quartz cavities. In brief, our detection limit ${\mathcal{I}}_{\text{LOD}}\approx 280\,\text{photon}\,{\text{s}}^{-1}$ corresponds to a source that would produce $17$ times less photons per unit time compared to one bacterium in an optimized ATP luminescence assay, and emit these photons inside inside a 2.5 L quartz cavity instead of the usual 0.1 cm^3^ sample volume of luminometers. Naively, if the same enzymatic luminescence efficiency and rate constants are considered, our detection limit is equivalent to $[\text{ATP}{]}_{\text{LOD}}\approx 23\,\text{zM}$ instead of $10\,\text{fM}$. Finally, knowing that one luciferase can typically produce $10 \,\text{photon}\,{\text{s}}^{-1}$^47,48^, our detection limit ${\mathcal{I}}_{\text{LOD}}\approx 280\,\text{photon}\,{\text{s}}^{-1}$ is equivalent to $28$ enzyme molecules or $46\,\text{yocto-mole}$, which means an enzyme concentration of 1.1 zM in a 43 cm^3^ Teflon cavity, or 19 yM in a 2.5 L quartz cavity.

### Effect of thermal radiation on luminescence detection

This extreme detection sensitivity raises the issue of the fundamental limit set by blackbody radiation^49^. In the empty cavity, the Kirchhoff’s law tells us that the very high reflectivity gives a very small emissivity equal to the cavity loss coefficient ${\epsilon }_{c}$, but the overall reflection losses are such that the exit port can be considered from the outside world as a grey body, i.e., an absorber with emissivity $1/2$. In addition, if thermal radiation is produced inside the cavity by a sample with an exothermic reaction, it will increase at equilibrium the output thermal flux. A careful analysis of the sensitivity of the camera to thermal radiation is presented in our companion paper (Fig. 5 in^5^). Given the extreme nonlinearity of the photonic spectral radiance of thermal radiation with the radiation temperature and the shape of the so-called UV catastrophe^50^, we found that our camera has its maximal sensitivity to thermal radiation at the ambient temperature for $\lambda =1050\text{ nm}$, despite a poor efficiency at that wavelength ($\text{QE}=1.9$ × 10^−2^).

As a consequence, the contribution of thermal radiation at the sample temperature (23 °C) amounts to a negligible fraction (1/40) of the noise floor of the camera, and it only exceeds that floor for *T* ≥ 48 °C (see Fig. 5 in^5^). From the point of view of temperature variations, the so-called noise equivalent temperature difference (NETD) typically amounts to 6 °C for *T* = 48 °C, and increases at smaller temperatures. This means that temperature difference less than 6 °C are not detectable in our experimental conditions. Consequently, all the variations that exceed the noise do correspond to variations of the luminescence. Nevertheless, for the sake of instrumental and physiological stability, the temperature was carefully controlled for the instruments (23 ± 0.5 °C) and the samples (23 ± 0.8 °C).

### Luminescence of H_2_O_2_ disproportionation

Thanks to the unprecedented sensitivity of our method, we evidence here that the disproportionation of hydrogen peroxide does indeed produce light in pure water, i.e., in the absence of the ingredients classically needed to make that reaction luminescent. The disproportionation of H_2_O_2_ is indeed a key reaction for a large number of assays, such as luminol-based detection systems, and it has been extensively characterized and carefully calibrated^6,8,13,26,31^. Although a number of investigators are aware of a background emission seen in the absence of luminol, it has only been reported once^36^, most likely because of its extreme weakness. This previous work reports on luminescence in the presence of salts, but results are given in arbitrary units, thus precluding a quantitative comparison with our work. They observed that the luminescence increases with pH with three major spectral peaks a $\lambda \approx 633$, $477$ and 703 nm. These peaks were later recognized to reflect radiative transitions of singlet oxygen which is the likely emitter^26,30^. Here in contrast, luminescence is seen in a chemically distinct environment with pure water equilibrated in the ambient atmosphere. Under the reasonable assumption that the luminescence spectrum here is similar with Kruk et al.^36^, it makes sense to consider that the quantum efficiency of our detector is close to its maximum ${\text{QE}}\approx 0.9$. This enabled us to translate the observed count rates into photon emission rates and rate densities, leading to the finding that the non-catalytic disproportionation of 88 mM H_2_O_2_ produces a photon emission rate density of ${\mathcal{J}}_{{\text{H}}_{2}{\text{O}}_{2}}\approx 73\,\text{z}{\mathbb{E}}\,{\text{L}}^{-1}\,{\text{ s}}^{-1}$, with a photon emission rate constant ${k}^{+}Y\approx 8$ × 10^−19^ s^−1^. Even though our observations beg for more detailed investigations, with spectral analyses and better environmental and chemical controls, we exhibit here what is likely the most elementary chemiluminescent reaction, with nothing else than ${\text{H}}_{2}{\text{O}}$ and ${\text{H}}_{2}{\text{O}}_{2}$. In that respect, this study should help investigate the fundamental interactions of water with light and oxygen, and the relation between the photochemistry of light absorption, and the reverse photochemistry we call chemiluminescence.

### Spontaneous luminescence of yeast cultures

Our observation of the spontaneous luminescence of *S. cerevisiae* yeast cells is congruent with a series of reports that clearly demonstrate that most cells and tissues including yeasts^15,18–22^ do spontaneously emit an ultraweak bioluminescence related to their oxidative metabolism and to basic mechanisms of radical-based chemiluminescence^15,19,22,23,51,52^. In this field, the most quantitative papers have essentially reported luminescence intensities in terms of photon fluxes with values in the range of $1$–${10}^{4}\,\text{photon}\,{\text{s}}^{-1}\,{\text{cm}}^{-2}$^19^. The light compression principle presented here could be used to amplify such weak fluxes from 2D samples. Indeed, if a 2D sample with a surface area ${\Sigma }_{s}$ is sitting on the inside wall of an optimized integrating cavity with a larger surface area ${\Sigma }_{c}$, $50\%$ of the light it emits is expected to exit the cavity, and the resulting exit flux is concentrated compared to the primary emitting surface, by a factor ${\Sigma }_{c}/{\Sigma }_{s}$ which has at best the same order of magnitude as $\rho /\epsilon$, i.e., a factor $1600$ with quartz powder cavities. However, some interesting biological samples such as liquid cultures are intrinsically characterized by a photon emission rate volume density rather than a surface flux, for which we provide here an absolute measurement as a function of time.

Our results show some experimental dispersion in terms of the time needed to reach the peak intensity, but much less dispersion in terms of how many photons are collected from each culture. The three plots shown on Fig. 4 (graph 4) lead to estimated total numbers of photons of 9.0, 11.5 and 11.9 × 10^6^ photons. Qualitatively, the luminescence peaks before the frequency of mitotic events reaches its maximum. This suggests that light production is associated with interphase rather than the effective cell division during cytokinesis, but this point remains to be further elucidated with the help of synchronous cultures, pharmacological treatments, and mutant strains. Quantitatively, the peak emission rate represents approximately $1\,\text{photon}\,{\text{s}}^{-1}$ for ${10}^{5}$ cells.

We should recall here that the loss coefficient ${\epsilon }_{Teflon}$ was measured in empty cavities and should be corrected for by the transmission loss coefficient ${\epsilon }_{s}$ of the sample to obtain the effective loss. A dedicated non-absorbing medium was used to minimize this effect. From the measured absorption and using the above theoretical derivation, we could estimate that the correction is less than 20–30% for $500<\lambda /{\text{nm}}<1000$. To fully take these effects into account and correct for stronger absorption, as well as for a possible change of the absorption caused by the yeast culture, the effective cavity loss coefficient $\epsilon (\lambda )$ should be evaluated over the whole spectrum in the presence of each kind of sample rather than as a constant of the empty cavity. In addition, the emission spectrum of the luminescence should be also determined and corrected for by the effect of $\epsilon (\lambda )$ to obtain the exact emission rate density $\mathcal{J}(\lambda )$ as a function of the wavelength. For both series of experiments, with ${\text{H}}_{2}{\text{O}}_{2}$ disproportionation and yeast luminescence, Teflon cavities were used instead of quartz cavities, because of purely technical limitations that await further developments. Teflon cavities are easier to build, more robust, simple to manipulate and wash. In contrast, building quartz cavities is a bit more complicated, and their walls must remain very dry and need to be thoroughly sealed off with extremely low absorption surfaces.

## Conclusion

Together with our work on detectivity-optimized EM-CCD detection^5^, the principle of cavity-mediated spatial concentration and dimensional reduction of light presented here is a major leap forward, because it simply grants an unprecedented access to the quantitative detection of ultraweak light emitted by extended 3D samples. Since light can be concentrated to produce a 2D flat Lambertian emitter with an arbitrary shape including a thin slit, our design also grants access to the spectral analysis of these extended light sources. A theoretical framework is proposed to define the optimal detection design, to predict its signal-to-noise ratio, and to analyze how it would scale under different design constraints. The unprecedented detection limit we obtain should help fundamental research efforts to quantitatively explore and understand ultraweak light emission phenomena such as chemiluminescence, delayed photoluminescence or afterglow, thermoluminescence, electro- and electrochemiluminescence, or Cerenkov radiation, in liquid or gas phases, or in transparent solids. It should also help to further investigate the connection between ultraweak bioluminescence and the oxidative metabolism and stress. Practically, by concentrating light instead of reducing the sample volume, a strong limit is removed for a number of luminescence-based detection technologies. Ulbricht cavities invented more than 100 years ago could be very helpful as efficient light concentrators, to develop novel light detection methods with unprecedented levels of sensitivity, with applications to non-absorbing solids, liquids, gases or aerosols of interest for basic research as well as environmental control, biohazard detection, medical diagnosis, or microfluidics.

## Methods

### Darkness control

A strict environmental control was implemented to reach the darkest possible conditions as described in our companion paper^5^. Briefly, all experiments were carried out inside a custom-made metal enclosure, covered by two layers of thick opaque black fabrics outside, with blackout optical fabric inside (Thorlabs, BK5), and located in a customized dark room. Complete darkness conditions used for all measurements practically refer to two criteria: (a) the background noise level could not be further reduced by additional light protections, and (b) the observed variations of the background detection level were non-monotonic. This last criterion is important because of the fact that countless objects (glass, plastic, paint, Teflon, fabrics, …) do produce a detectable delayed photoluminescence when transferred in the dark. This unwanted luminescence sometimes relaxes over days before a stable “background” level is reached. As a consequence, all the objects belonging to the set-up are kept in the dark or transported in dark conditions as much as possible. Because of this significant delayed photoluminescence, we used quartz instead of glass containers as much as possible.

While the detector temperature was kept stable at − 70 ± 0.05 °C, the temperature in the dark enclosure was kept at 23 ± 0.5 °C. Integrating cavities were home-made, from Teflon or compressed quartz powder. Cavities machined from Teflon were made of two parts, a container and a lid as shown on Fig. 2a. The inside volume was a 43 cm^3^ cylinder with radius *R*_*c*_ = 19 mm, height $h=2{R}_{c}$, and 25 mm thick wall. The output port was drilled as a 4.4 mm diameter hole in the lid.

### Integrating cavities

Quartz cavities were made from synthetic amorphous silica powder (ZANDOSIL 30, Heraeus), which was compressed and baked as first indicated by Cone et al.^29^. Cavities were made of two parts, similarly to the Teflon cavities: a container and a lid as shown on Fig. 2a. The inside volume was a cylinder with radius *R*_*c*_ = 23 mm and height h = 20 mm. The albedo $\rho$ of our integrating cavities was measured using the well-known ring-down method^30^, based on assessing how fast a short light pulse exponentially fades out inside the fully closed cavity. The exponential decay time ${\tau }_{r}$ obtained is compared to the average travel time ${\tau }_{ch}=c{\overline{l}}_{ch}$ of a photon across the empty cavity, where ${\overline{l}}_{ch}$ is the average chord length. The well-known mean-chord-length property^28^ states that ${\overline{l}}_{ch}$ only depends on cavity volume ${V}_{c}$ and its enclosing surface area ${\Sigma }_{c}$ regardless of the shape as ${\overline{l}}_{ch}=4{V}_{c}/{\Sigma }_{c}$. A simple statistical argument on the chained probability for a photon to be absorbed after $n$ reflections leads to $\rho ={e}^{-{\tau }_{ch}/{\tau }_{r}}$.

Practically, the albedo was controlled by measuring the broadening of a 670 nm and 40 Hz picosecond laser (PC-670 M and PDL-800B, PicoQuant), with 120 ps and 2 mJ pulses. A Time Correlated Single Photon Counter (TCSPC PicoHarp 300 system, PicoQuant) was used with a SIA 400 attenuator/inverter module (time bin width 4 ps, dead time less than 95 ns, maximum sync rate of 84 MHz, 16 bits, 2 channels). The albedo of the quartz cavity was found to be ${\rho }_{\text{quartz}}=0.9994=1-6\times 10^{-4}$, and ${\rho }_{\text{Teflon}}=0.991=1-90\times 10^{-4}$ for Teflon cavities. While the albedo obviously depends on the wavelength, Cone et al.^29^ found similar values with 0.99919 at 532 nm for quartz.

### EM-CCD cameras and optical set-up

Two similar EM-CCD cameras were used, model HNü512 (Nuvu, Montreal, Canada)^53^. Their main nominal characteristics are: 512 × 512 pixels with a 16 × 16 µm^2^ area each, spectral range from 250 to 1100 nm with quantum efficiency better than 90% at 600 nm and $0.8\le \text{QE}(\lambda )\le 0.9$ for $400\,\text{nm}\le \lambda \le 750\,\text{ nm}$, back-illumination with inverted mode operation (IMO), thermo-electric cooling down to − 85 °C, EM-gain from 1 to 5000, EM register pixel capacity of 8 × 10^8^ electrons, read-out noise with multiplication < 0.1 *e*^−^. The cameras were operated in binary counting mode with an EM-gain of 3000, a pixel frequency of 10 MHz, and cooled at − 70 °C. The actual noise figures were measured for each pixel and averaged over the whole detector for both cameras, as explained in^5^. For cameras #1 and #2, respectively, the clock-induced-charges noise was measured to be 1.4 × 10^−3^ and 1.13 × 10^−3^ e^−^ pixel^−1^ frame^−1^, and the dark current was 5.2 × 10^−4^ and 4.1 × 10^−4^ e^−^ pixel^−1^ s^−1^.

The camera sensor was conjugated to the cavity exit port with a high NA (NA = 0.29) lens (KPA040, Newport) with *F/*# = 0.6, ∅ = 40 mm and a 22 mm focal length. Practically, this lens was adjusted so that the Teflon cavity exit port produced a 8 mm diameter disk on the 8.16 × 8.16 mm^2^ sensor, with a magnification ratio $M=0.94$. A Σ_*d*_ = 0.46 cm^2^ central disk was considered as the detection area on the sensor. All software control and data processing were achieved from a home-made Python interface software using the NuVu acquisition library.

### Chemiluminescence experiments

Chemiluminescence experiments with hydrogen peroxide were conducted inside Teflon cavities designed as described above, in the dark enclosure described in our companion paper^5^ with controlled temperature 23 ± 0.5 °C. The sample was saturated with air using a 50 mL min^−1^ flow through a transparent tube (0.5 mm internal diameter Tygon). Light was collected on the detector from the circular region (Σ_*d*_) conjugated to the exit port Fig. 2a. The region outside of the disk was used to monitor the level of stray or background light that did not come from the cavity exit port. Teflon cavities were kept in the dark for at least two days, to minimize background variations due to their delayed photoluminescence. Teflon cavities were cleaned as follows: washed with water and soap, dried, washed with chloroform, acetone, ethanol and isopropanol, autoclaved and put under oxygen plasma (50 W, 0.5 bar) during 2 min. For all experiments, solutions were also kept in the dark for at least 1 day.

Solutions inside the quartz cavity were contained in custom-made quartz containers, to avoid the slowly decaying photoluminescence of glass, and containers were washed as described above. Samples were injected into and removed from the cavity by remote syringes, through transparent tubes inside the cavity, to minimize light absorption and the resulting reduction of the albedo. Outside of the cavity, these tubes were instead connected to dark tubes to limit light transmission from outside sources.

For experiments with hemoglobin, we followed the protocol of Lee and Seliger^8^. Human hemoglobin (H7379-1G, Sigma) was prepared as a 0.5 mg mL^−1^ solution in water and diluted to 1*/*10 in 10 mL samples. We used milliQ water, filtered with 0.22 µm filters. Solutions of H_2_O_2_ were prepared extemporaneously from a 30 wt% solution (sigma H100S-100 mL). All solutions are prepared before the experiment, kept in the dark at least for 12 h and protected at all times from light exposure by aluminium foils. All experiments were carried out at atmospheric pressure.

### Yeast experiments

Yeast strain BY4742 was a gift from Basile Jacquel (IGBMC, Strasbourg, France), and liquid cultures were grown in a 6.9 g L^−1^ Yeast Nitrogen Base without amino acid (CYN0402, Formedium) completed with 2.002 g L^−1^ synthetic Drop Out mixture (DSCK1000, Formedium) and 20 g L^−1^ glucose. The medium was autoclaved, filtered (0.22 µm, Nalgene), complemented with glucose and kept at room temperature in the dark. Light measurements were performed with both cameras and two similar Teflon cavities. After an initial time interval used to assess the background stability and level of the 20 mL culture medium, the culture was seeded with a 1 mL volume of yeast from a 15 days old stationary phase culture. Seeding was performed remotely using a transparent tube (0.5 mm internal diameter Tygon), without breaking the darkness, and with continuous recording of light emission with a *τ* = 120 s exposure time. A third culture was grown in the exact same conditions outside the dark enclosure, but in a twice larger volume, for the sake of easily counting the cell density along time. Cell densities were manually assessed (Incyto, C-Chip, DHC-N01) for all samples at the end of the experiment, and no statistically significant difference was found between them. Cultures were oxygenated by a 80 mL min^−1^ flow of filtered air through a transparent tube (0.5 mm internal diameter Tygon). The temperature was monitored for all cultures by RTD probes (HSRTD-3-100-A, Omega) and thermocouple probes (TWHS-K, Thermosense Direct) coupled to a temperature controller (PTC10, Stanford Research System). Temperature remained stable with *T* = 23 ± 0.8 °C. Probes were located inside the integrating cavities and inside the external control sample. All quartz glass-ware and integrating cavities were cleaned as described above.

## Supplementary Information

Supplementary Legend.

Supplementary Figure 1.

## Data Availability

The data that support the findings of this study are available from the corresponding author upon reasonable request.
