# Supplementary material for: Stochastic light concentration from 3D to 2D reveals ultraweak chemi- and bioluminescence
Source: Sci Rep. 2021 May 11;11:10050. doi: 10.1038/s41598-021-88091-0 (PMC8113247; doi:10.1038/s41598-021-88091-0)
Supplement: Supplementary file 1 — Supplementary Legend. [file 41598_2021_88091_MOESM1_ESM.docx]

**Supplementary Material – one single figure & legend**

Stochastic light concentration from 3D to 2D reveals ultraweak chemi- and bioluminescence

Khaoua et al.

**Legend**

**Overall set-up**

The detection set-up sits inside a custom-made metal enclosure (0.8x0.8x0.8 m^3^) protected with extra layers of thick opaque black fabrics and located in a customized dark room. The notion of “complete darkness conditions” practically refers to two criteria: (*a*) the background noise level could not be further reduced by additional protections from light, and (*b*) the remaining variations of the background detection level were non-monotonic. All elements are kept in the dark or transported in dark conditions as much as possible. While the detector temperature was kept stable within −70±0*.*05 ^◦^C, the temperature in the dark enclosure was kept at 23±0*.*5 ^◦^C. Home-made integrating cavities (Teflon or compressed quartz powder) were coupled to the camera by a lens.

**Cavity + optics details**

Cavities were made of two parts, a container and a lid. The inside volume was a 43 cm^3^ cylinder with radius *R_c_*=19 mm, height h=2R_c_, and 25 mm thick walls. The practical design followed (a) the prescription of our theoretical model to achieve the best sensitivity to detect photons emitted by the sample, given in units of number of photons per unit time per unit volume, and (b) the results of our work to optimize the detectivity of the EM-CCD (see Khaoua et al. Scientific Reports, January 2021).

Practically, the cavity output port for Teflon cavities was drilled as a 4.4 mm diameter hole in the cavity lid, to match the sensor detectivity. A high numerical aperture lens (NA=0.29) was used to optimize the flux density collection efficiency, with *F/*#=0*.*6, ∅=40 mm and a 22 mm focal length. The lens couples the exit port with the camera sensor with a magnification close to unity (M≈0.94), and the flux on the sensor represents NA^2^/M^2^ ≈ 9.5% of the flux at the exit port. Finally, the camera has a 0.67 cm^2^ EMCCD sensor with 512^2^ pixels operated in the binary photon counting mode.

**Detection limit**

Using this design, from the photons produced in the cavity, 50% of exit through the hole and 4.2% reach the detector. Given the sensor’s detectivity (0.015 photon^-1^s^1/2^cm), the detection limit (for SNR=3) is 0.11 photons/s.cm^3^ (= 110 photons per second per Liter) for quartz powder cavities, and 6.6 photons / s.cm^3^ for Teflon cavities.
