# Supplementary figures and images for: Stochastic light concentration from 3D to 2D reveals ultraweak chemi- and bioluminescence
Source: Sci Rep. 2021 May 11;11:10050. doi: 10.1038/s41598-021-88091-0 (PMC8113247; doi:10.1038/s41598-021-88091-0)

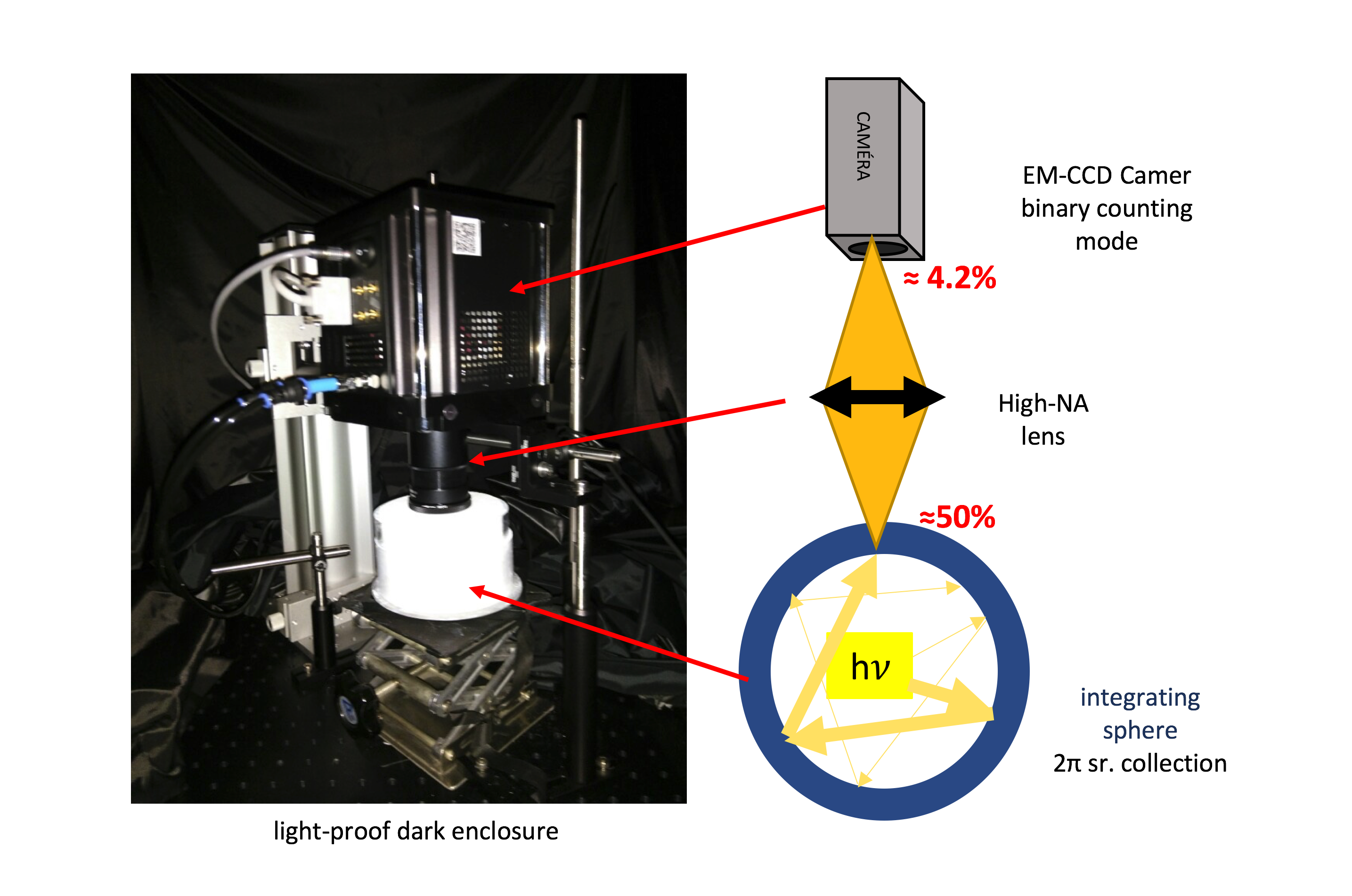

Supplement: Supplementary file 2 — Supplementary Figure 1. [file 41598_2021_88091_MOESM2_ESM.png]
